# Approaches to address the gap in research on energy use and racialization in the UK

**DOI:** 10.1007/s12053-025-10390-6

**Published:** 2026-01-08

**Authors:** Uttara Narayan, Sarah Higginson, Nick Eyre

**Affiliations:** 1 School of Environment, Education and Development, University of Manchester, Oxford Road, Manchester, M13 9PL UK; 2 Faculty of Architecture, Building and Planning, and Student Member of Life Course Centre, University of Melbourne, Melbourne, VIC 3010 Australia; 3 Energy Demand Research Centre, University of Birmingham, Birmingham, AL B15 2TT USA; 4 Environmental Change Institute, University of Oxford, South Parks Road, Oxford, OX1 3QY UK

**Keywords:** Energy use, Racial justice, Energy justice, Racialization, Just energy transition, Racialized social system approach

## Abstract

This paper shares findings from research at the intersection of energy use and racialization — an under-researched topic in the UK. Existing literature can be further strengthened by emphasising the energy use experiences of those who are racially disadvantaged, the role played by structures in contributing to or addressing these disadvantages, and what achieving racial justice can look like, in the UK’s context. We sought to understand the reasons for this gap and identify potential approaches to address it in the UK, through semi-structured interviews, whose responses were analysed thematically and validated through a workshop. Participants for the semi-structured interviews (n = 27) and workshop (n = 45) were identified among energy and social researchers and practitioners in the UK. Interview questions explored reasons for the dearth in research, understanding the current state of energy and racialization research (at an experiential and structural level), and aspiring for racial justice in energy research (through a pluralistic definition of racial justice). Thematic qualitative analysis of the interviews, that were further validated by the workshop, revealed the following areas for further investigation—(a) Energy services where people demonstrably experience racialized disadvantages, namely in the built environment, transport, health, and food; (b) Processes through which racially disadvantaged people are disproportionately impacted by how the energy system is governed, especially with respect to norms around how energy use is managed, trust and acceptance of low-carbon technologies, and access to energy advice services; and (c) Methods to strengthen the evidence’s explanatory power in a non-extractive manner. The paper concludes with a call to further research about the diverse energy practices and needs of racially disadvantaged people, thereby challenging assumptions around an archetypal low-carbon energy consumer, and its implications for energy justice in the path to decarbonise.

**Supplementary Information:**

The online version contains supplementary material available at 10.1007/s12053-025-10390-6.

## Introduction

Consequent to the protests after George Floyd’s murder and the global resurgence of the Black Lives Matter movement in 2020, the Centre for Research into Energy Demand Solutions (CREDS) recognised that there was a dearth of research at the intersection of energy use and racial justice in the United Kingdom, where CREDS is based. In response, CREDS commenced scoping research to understand this gap and aimed to build towards a research agenda to understand the relationship between energy use and racialization, and further racial justice in energy research (Higginson & Fadare, 2022; Narayan, 2023). This paper is an outcome of that scoping research.

### *The relationship between energy use and racialization*

While it is important to understand how, when, what and how much energy is used, it is equally important to understand *who* uses this energy, and how they are able to use it. Understanding the relationship between energy use and racialization is the focus of this paper, for two reasons—firstly, energy demand was the research focus of CREDS, and secondly, the main contributors of energy demand, the built environment and transport (DESNZ, 2023), demonstrate evidence of significant racialized disadvantages.

Research by Zewolde et al. (2020) on built environment, Neely and Samura (2011) on space, and Rutten (2020) on planning have initiated conversations to consider the role of race in these areas. Camargo (2020) and the Race Equality Foundation have highlighted how healthcare is racialized, and the relationship between energy, built environment and well-being is demonstrated for the USA by Hernandez (2016) and Lewis et al. (2020), and England, UK by Huebner et al. (2022). Within the built environment, emerging research exhorts the need to build further evidence on racialized disadvantage around (a) housing (Danewid, 2022; D'Souza & Khan, 2021; Gulliver, 2017; Raslan & Ambrose, 2022; Willis, 2018), especially in the private rented sector (Bouzarovski et al., 2022), (b) access to green spaces (The Ramblers, 2020), and (c) heating (Anguelovski et al., 2025) and cooling needs (Kidwell & Ogunbode, 2022). Similarly in transport, Schwanen (2018) has called for decolonising transport research, Gates et al. (2019) observed racialized disparities in daily commute, and Mattioli and Scheiner (2022) consider air travel practices and preferences in relation to passengers’ ethnicities and migration histories.

Processes to manage energy use also hold the potential to be racialized. Phillips and Petrova’s (2021) research from South Africa demonstrates how systemic factors such as political processes contribute to gendered and racialized energy vulnerabilities. Hodges et al.’s (2022) research in Bristol with South Asian communities discovered that multigenerational households’ practices around managing energy use are quite distinct from those that are assumed to be the norm while identifying thresholds for appropriate energy use. Similarly, there is emerging evidence of barriers that different marginalised groups face when attempting to access energy advice, as Bouzarovski et al. (2022) demonstrate for recent immigrants, and Forster et al. (2019) and Sovacool and Del Rio (2022) for the Traveller community. Owen et al.’s (2023) work challenges assumptions around ethnic minority groups being ‘disengaged’ from participating in low-carbon initiatives, and Lennon (2017) displays how technical energy stakeholders tend to take a paternalistic view towards marginalised people. Newell (2021) and Lennon (2021) highlight that this challenge is ubiquitous to the energy system and how it is governed. Incremental attempts to address racialized disadvantages are insufficient, and require a systemic approach that considers the energy system as well as the social system.

### *Energy use within a racialized social system*

While attempting to understand the history of racial formation and racialized interactions in the UK, the authors realised that categories such as ‘race’, ‘ethnicity’, or ‘ethnic minorities’ can be limiting in understanding *why* someone was affected in the way they were able to use energy. This compelled us to understand how racial meaning (and hierarchy) becomes attached to people’s identities, thereby affecting their ability to interact with systems that might generally be assumed to not be inherently racial, such as the energy system. This is broadly how racialization is defined in the literature, and therefore, we recognised the need to investigate the relationship between racialization and energy use in order to understand its implications for energy and racial justice (Gonzalez-Sobrino & Goss, 2019; Meghji, 2022; Glynn, n.d.). Framing this as ‘racialization’ also allows us to understand how such group identities are formed (thus demonstrating the dynamic nature of such constructs) thereby impacting the way people interact with each other and society. Therefore, it allows for analytical possibilities of understanding how different social groups interact with, and are impacted by social structures, that does not essentialise their behaviours and practices. For example, it allows for an understanding of the experiences of the Traveller community, a historically marginalised group and economically precarious recent immigrants to the UK, in a way that might not be sufficiently captured by categories such as ‘race’ or ‘ethnicity’.

The conceptualisation of racialization is contributed by Critical Race Theory (CRT) (Gonzalez-Sobrino & Goss, 2019). CRT is concerned with demonstrating the structural nature of racialized disadvantage. It demonstrates that despite the seemingly legal and institutional ‘gains’ made in light of the Civil Rights Movement in the United States, the indicators of material improvements and well-being such as improved life expectancy, social mobility, etc. remained significantly different between social groups, thereby diagnosing the cause of such differences to be structural. Given CRT’s emergence in a specifically US context, there has been some hesitation to apply it elsewhere. There has also been criticism of CRT scholars being ‘methodologically nationalist’ because of their exclusive focus on the US. However, in the UK, whose history of racialization is distinct from that of the US where CRT originated, Ali Meghji’s scholarship has contributed to understanding its application, especially in a post-Brexit UK, particularly through the Racialized Social System approach (Meghji, 2020, 2022).

Conceptualised by Eduardo Bonilla-Silva (1997), the racialized social system is one where social, political, economic, cultural and psychological outcomes and resources are partially allocated with respect to racial position. The allocation is only partially attributed to racial positions because, the approach acknowledges the intersection of racialization with gendered and classed social divisions. The racialized social system’s focus on the systemic allows for the understanding that participating in the reproduction of the racialized order is inevitable, irrespective of identity, positionality and values (Bonilla-Silva, 2021). This approach recognises the social system as racialized and therefore omnipresent, much like capitalism and patriarchy. It acknowledges the material nature of racialization that benefits some groups and disadvantages others in realms perceived to be ‘race-neutral’.

The racialized social system approach is useful for our enquiry into the relationship between racialization and energy use, because of its ability to link the wider structures of society and the energy system with everyday interactions and experiences, through its conceptualisation of the racialized interaction order. The concept allows for an interpretation of the energy system as being situated within a wider racialized social system (i.e. society) wherein the energy system influences and is in turn influenced by society and everyday interactions within it.

We observe that the energy system is at the meso-level of the racialized social system—operating within society in order to provide important services that are meant to support users’ needs for thermal comfort, cooking, etc. This allows us to understand it as a ‘social mechanism’ within society that impacts, and is also influenced by, everyday interactions (such as an energy user’s experience with paying bills or seeking advice to manage energy use in order to function in society). Social mechanisms provide explanatory rather than causal descriptions (Meghji, 2022). Therefore, it is instructive to analyse the energy system as a ‘racialized organisation’ situated at the meso-level.

At the societal level, in realms that are often assumed to be ‘race-neutral’, such as the economy, insurance markets, healthcare, there are varying degrees of evidence of racialization. COVID-19 made explicit the racialized inequalities in healthcare, in terms of risks as well as outcomes (Camargo, 2020; Public Health England, 2020). The UK’s Office of National Statistics (2022) data revealed that 47% Black or Black British adults were unable to cope with the cost of living crisis in comparison to 28% White British adults, and austerity measures reduce their access to welfare, and further racially disadvantage immigrants who are made ineligible for state welfare support (Bhambra & Holmwood, 2018; Bouzarovski et al., 2022; Edmiston et al., 2022). As Hodges et al. (2022) note, there are invisible barriers to how racialized groups access these benefits, including, language barriers to obtaining consumer advice, as English might not be their first language and a hesitation to avail state benefits even when they are eligible. Households categorised as ‘ethnic minority’ tend to live in more overcrowded and dilapidated housing conditions (The Health Foundation, 2023), thereby experiencing fuel poverty more severely. Research from the Joseph Rowntree Foundation, showed that households headed by those of Bangladeshi heritage are more likely to be at risk of living in an inadequately warm dwelling, and households headed either by those who are Black or of Bangladeshi heritage are most likely to fall behind on paying bills (Matejic & Earwaker, 2022). Furthermore, one in three homeless people in London are people of colour (Gulliver, 2017). Ethnic minority communities demonstrate a greater reliance on public transport, and tend to be situated farther away from access to essential services, increasing their risk of transport poverty (Gates et al., 2019; see Casado-Díaz et al., 2022 for evidence from Spain and bunten et al., 2022 for Philadelphia in the US). Research from Citizens Advice discovered that there is an ‘ethnicity penalty’ whereby areas with significant ethnic minority populations tended to pay higher car insurance premiums (Cook et al., 2022). Recent immigrants (those living in the UK for less than five years) with an ethnic minority background might be doubly disadvantaged— Bouzarovski et al. (2022) discovered that 75% of recent migrants live in the private rented sector which is known to have some of thr poorest energy efficiency ratings. These are some examples of how different sectors might unequally distribute benefits and risks based on people’s racialized positions.

There is broad consensus on the dearth of research surrounding the relationship between racialization and energy use, specifically in the UK. Some of the reasons behind the persistence of this gap, include—

*Availability of data and its appropriate classification in a non-essentialising manner*: Ahmadzadeh (2021) outlines the nuances involved in using terminologies like race and ethnicity and provides guidelines on the most appropriate terms (she recommends the use of ancestry or heritage). Existing literature conceptualizes racialization insufficiently. It predominantly focuses on static characteristics such as ethnicity or race, rather than how people and communities are racialized, and how that may disadvantage their energy use. The analyses consider race or ethnicity as an additive characteristic rather than looking at the multiplicative relationship between race/racialization and other social characteristics like gender and class. Many larger-scale (usually national level) quantitative research appear to be possible because of existing public surveys, such as the Residential Energy Consumption Survey (RECS), in the USA, and the Household, Income and Labour Dynamics in Australia Survey (HILDA), in Australia, and the public infrastructure to collect and manage them but it might be worthwhile for these surveys to also revisit these terminologies.

*Insufficient diversity within the research community*: D'Agostino et al. (2011) discovered that energy research between 1999–2008 was dominated by male authors from North America with a science or engineering background. UKERC (2022) is undertaking research to understand the experiences of ethnic minority professionals in the UK to inform approaches to diversify the energy sector.

### *Understanding racialization in relation to the energy system*

The literature on conceptualising racial justice with respect to the energy system broadly falls under three categories—(a) an extension of the energy justice tenets, namely distributional, procedural and recognition justice (Bouzarovski et al., 2022; Jenkins et al., 2016, 2020), (b) drawing on the historical contexts of colonialism and racial capitalism in establishing these energy systems (Bhambra & Newell, 2022; Kothari, 2006; Lennon, 2021), and (c) framing around accessing opportunities and ease of using services (Bouzarovski et al., 2022; Creutzfeldt & Gill, 2021; Forster et al., 2019). Articulating a coherent research vision around any of these categories requires explicitly anti-racist approaches with respect to data collection (Ahmadzadeh, 2021), effective engagement between researchers and those who are the subject of research (Blakelock, 2021; Creutzfeldt & Gill, 2021; Devine-Wright & Ryder, 2024), and robust conceptualisation of social theories in the context of energy demand (Bouzarovski, 2022; Cannon & Chu, 2021; Sovacool et al., 2023) with corresponding analysis, and principles of political education and engagement (Kapoor et al., 2022; Pulido, 1996).

From the literature it emerged that, in the context of the UK, we did not know enough about:

- The energy use experiences of those who are racially disadvantaged,
- The interaction between structures and experiences and the role structures play in contributing to or addressing these disadvantages, and
- What achieving racial justice could look like.

In recognition of this research gap, the paper sought to understand why these gaps persist, how to address them, and how that might potentially contribute to racial justice.

## Methods

Given the emergent and exploratory nature of this enquiry, the study undertook an explicitly qualitative approach. It sought to investigate the explanations for the dearth of research in the context of the UK and explore the applicability of the concept of racialization (instead of using limiting terminology like BAME or race and ethnicity), therefore lending itself to a semi-structured interview-based research method.

**Fig. 1:**
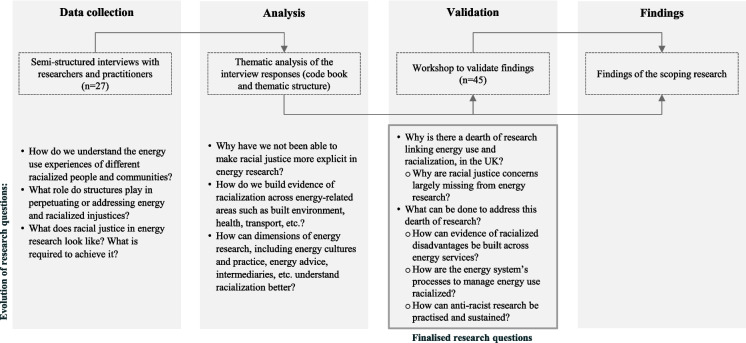
Methods and the evolution of the research questions

The research is based on 27 semi-structured interviews with researchers and practitioners engaged in energy and social research predominantly in the UK. Their interview responses were analysed thematically, using NVivo. The emerging themes were shared for feedback and validation with the wider community of energy and social researchers and practitioners through an online workshop, that helped finalise the findings. The purpose of the workshop was to validate the findings from the interviews and not to generate new data. This sequence also helped us refine our research questions, which during the data collection phase, were centred on addressing the research gaps from the literature that informed our semi-structured interview questions. During the thematic analysis, the questions emphasised the themes including the built environment, energy advice, etc. that emerged from the interview responses. And finally, the workshop helped synthesise these into understanding the existing challenges, and actions to address the challenges by focusing on the themes and methods—this is used to structure the findings. This iterative approach is presented in Fig. 1 below:

### Semi-structured expert interviews

Semi-structured interviews (n = 27, of whom at least eight respondents had identified themselves as experiencing racialized disadvantages) were conducted among researchers and practitioners working on energy use and social research, across Europe but primarily in the UK (nine respondents conduct research beyond the UK). The respondents were identified through their association with CREDS and authorship of publications on energy use and some aspect of racialization (such as exploring experiences of those identified as ethnic minorities, first generation immigrants, asylum seekers, etc.), followed by snowball sampling. Given the emergent nature of research, and drawing upon the available literature, the respondents were asked the following questions:

1. How do we understand the energy use experiences of different racialized people and communities?
2. What role do structures play in perpetuating or addressing energy and racialized injustices?
3. What does racial justice in energy research look like? What is required to achieve it?

### Thematic analysis

Interview transcript data were analysed thematically, using NVivo. A thematic analysis was deemed an appropriate analytical approach to meet the research objectives of improving our understanding of the research gaps and identifying focus areas to further anti-racist energy research, in an emerging research field (Kiger & Varpio, 2020). The coding tree was developed inductively from the interview data and further triangulated against the insights from existing literature on energy use and racialization (details of the coding definitions mapped to relevant literature can be found in Supplementary Material #2). This approach strengthened the insights from the review. For instance, mobile work as a dimension of enquiry did not emerge from the literature, but the racialization of platform economy-based mobile work in the UK was discovered during the interviews. The workshop was designed around the themes that emerged from the analysis of interviews.

### Workshops

Preliminary results were presented in a three-hour online workshop in April 2023 to obtain feedback on the emerging findings. The workshops were not intended for data collection purposes, but to validate the findings emerging from the thematic analysis. The workshop included 45 participants from research, academia and industry, engaged in energy and/or social research in the UK and Europe Some of the workshop participants had already participated in the interviews. Structured as an interactive session, the workshop required the audience’s participation in virtual break-out sessions that were organised around the following questions that were derived from the thematic analysis. Participants were given the choice of selecting a specific group at the time of registering for the workshop. Some of the interview respondents, whose research was proximate to understanding racialization and energy use were requested to participate in the workshop as facilitators. The facilitators role included directing the discussion in the breakout groups, and summarising the discussions during the plenary:

- Why have we not been able to make racial justice more explicit in energy research?
- How do we build evidence of racialization, across energy-related areas such as the built environment, health, transport, etc.?
- How can dimensions of energy research, including energy cultures and practice, energy advice, intermediaries, etc. understand racialization better?

**Fig. 2:**
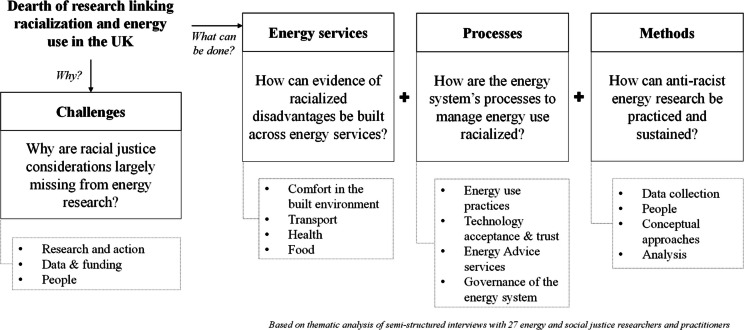
Structure of the thematic analysis

Based on discussions during the workshop, the preliminary findings were developed further, and the final thematic structure (Fig. 2 in the next section) that the findings are based upon were developed through the following research questions:

- Why is there a dearth of research linking energy use and racialization, in the UK?
  o Why are racial justice concerns largely missing from energy research? (Challenges)
- What can be done to address this dearth of research?
  o How can evidence of racialized disadvantages be built across energy services? (Energy services)
  o How are the energy system’s processes to manage energy use racialized? (Processes)
  o How can anti-racist research be practised and sustained? (Methods)

## Findings

The analytical enquiry begins with a recognition of the dearth of research linking energy use and racialization, in the UK. The analysis first sought to understand the challenges around why this research gap persists. In the spirit of articulating a research agenda that might address this gap, the remaining three analytical enquiries adopt a more action-oriented approach, beginning with (1) the energy services where evidence needs to be built and strengthened, (2) understanding the processes within the energy system that might perpetuate racialized disadvantages, and (3) ways to practice and sustain anti-racist principles in energy research. This section is organised around this logic, as shown in Fig. 2.

### *Challenges*

This theme sought to answer the question—why are racial justice considerations largely missing from energy research? The thematic analysis discovered that the persistence of these challenges can be attributed to—(a) the nature of this research and the responsibility to take action, (b) the availability of suitable data and funds to collect such data, and (c) appropriate representation among people who conduct the research as well as those who are considered subjects of such research.

#### Research and action

Linking a systemic issue like racialization to specific empirical studies on energy use presents a challenge of bridging the different scales concerning contemporary lived experiences with historic structural factors. In circumstances where evidence already exists, it is important to translate the diagnoses into actionable interventions that can be embedded in practice:*“We need more intervention studies in social research. What are the impacts of trying to change something? Because we often just correlate and act on the results..*” (AES4^1^). There is an added complexity that conducting research of this kind presents, as observed by AE4: “*There are probably some quite clear reasons relating to research that’s like kind of treading on sensitive ground, whether it's because you know, race and ethnicity falls under… kind of like… special category data under GDPR. I think, having that extra level of thought and care given to collecting data of that kind is probably a big barrier… like I suspect people just explicitly don't collect it for that reason to make their lives easier.”*

#### Data and funding

Racialized disadvantages might appear evident in certain aspects of society, thereby making remedial responses such as affirmative action, plausible. However, with respect to energy use it is not obvious, unless the relationship between structural issues and everyday experiences is consciously investigated, such as by proactively considering those who might be overlooked in the energy transition. AES4 corroborates this: “*It would mean that we put equal emphasis on the voices of ethnic minorities, as of the typical white household. So that we collect as much data from them as from other parts of the population. And I think that’s the part where we have spectacularly failed in the past*.” Some datasets are too small to conduct statistically significant analysis even with adequate de-identification. Furthermore, many respondents opined that limited funding opportunities result in uncoordinated and restricted studies with limited explanatory capacity that might be unable to comprehensively address these research gaps:*“There are small amounts of money out there to do research on this issue. But little pots of money mean that researchers pick up small scale studies.*” (AES8).

#### People

Lack of diversity in the energy research communities also contributes to the perception that the energy transition is a ‘white, middle-class, predominantly male’ concern—a refrain observed throughout many of the interviews. “*You know, in this country, the energy system and the energy efficiency community is very much, you know, a white person's environment and there's very, very few people who don't fit that picture who are working, you know, developing the technology, working in the field.*” (PES1) Labelling those outside this demographic group as ‘hard-to-reach’ or ‘difficult-to-engage’, places the onus of action on already marginalised people rather than the system. Some of the reasons for the underexploration of the link between energy efficiency, housing and racial justice include (1) the absence of a clear contact person representing private tenants (like a housing or a tenants’ association representative): “*And I think the private sector to me is a much bigger issue because it's just a lot less regulated, but it's also more difficult to research because you don't have like a clear contact person. We tend to do lots of studies with social housing providers because you have like a contact person, there is a housing association and then there's a tenants association. So it's quite easy to go in there and do some research. Well, comparatively easy to reach in there to research. But when it comes to the privately rented sector, which is the worst sector, like the poorest housing quality in this country in general, it's much harder to research because there's just no contact person.*” (AES4), (2) the research design might not be considerate to the needs of people and how they could benefit from the research and hence, they do not have an incentive to participate. AES9 raises this concern: “*It is important to design research questions in more experiential ways that is sensitive to the respondents’ experiences, which are not always easy in these circumstances.*”, and (3) many immigrant tenants are vulnerable to the UK Home Office’s hostile environment policy, and might be reluctant to candidly share negative experiences: “*…the kind of changes to the welfare system that have occurred in the UK over the last decade and a lot of that has had an explicitly kind of racialized dimension to it and it's about kind of, yeah, hostility really towards migrants*” (AES7).

### *Energy services*

In this section, we explore ways to strengthen evidence concerning racialized disadvantages experienced across energy services. The analysis identified four areas to build and strengthen evidence on racialization and energy use—(a) comfort in the built environment (that includes housing, access to green spaces, and heating and cooling), (b) transport (that not only concerns daily commute, but also air travel and mobile work), (c) health, and (d) food.

#### Comfort in the built environment

Whilst respondents recognised housing as a significant component to investigate, they also reflected that it is important to expand the scope beyond the buildings that people inhabit, to consider the broader built environment. Poor housing security and poor-quality building stock makes energy demand reduction measures (like insulation) less appealing, leading to higher energy use and costs. This is especially a challenge in the private rented sector*—“If you’re in the private rented sector, it’s gonna be really hard to get panels on your roof or any of these things that you need to be able to shift your demand and still have access to the energy services that you need, especially because your tenure is short or uncertain.*” (PES2) The location and quality of housing is determined by several socio-economic factors, which may determine the type of contract that tenants hold with their energy supplier. Many local authorities, who used to have a more grounded perspective of the buildings in the neighbourhood that needed insulation and the corresponding ability of residents to fund these interventions through their local area action plans, no longer have those powers—"…*a few local authorities had some really good schemes, but their funding has been squeezed and squeezed and very few of them can keep that going now, or if they can, it's very reduced. And in place of that you tend to have sort of online things where you key in your problem and you get a generic type of answer and yet energy use is such a personal thing.*” (AE3).

#### Transport

The interview responses explored the relationship between transport, energy and racialization beyond the daily commute, and reflected on nuances pertaining to air travel, mobile work, and accessibility of low carbon travel modes, such as active travel. The analysis revealed that the ability to choose transport modes, and exposure to longer commute time are racialized (thereby increasing the risk of exposure to extreme temperatures and air pollution). Low-emission and active travel are not equally accessible to all members of the population—according to AE1: “*Transport vulnerability is very important in this country. A Prime Minister once said that–At 29, if you're traveling by bus, you haven't really made it, and you should question your life choices. Of course, we are now promoting cycling in this country, but how many people can afford it? And you have cycle to work schemes and everything, but like who, who benefits from the them?*”. Living close to public transport increases property prices, crowding out those less able to pay, who are ironically in greater need of public transport. As AES3 noted, “*I think… living near a tube is a very huge indicator of… like, your house price and sort of more wealthy, commuters sort of end up near there, you know*”. Air travel also varies between different social groups —*“Even families on really low incomes will set aside money to do that international flight every year to go back and see family. That is very different in comparison to a flight to a European weekend getaway.*” (AE3). Another dimension that emerged from the interviews, and was not explicit in the literature was mobile work, often undertaken by racially disadvantaged people exposed to precarious contracts and working conditions. They provide mobility services as van and food delivery drivers, especially in the context of digitalised platform economies—“*Thinking about mobile work is a clear entryway into thinking about racialized mobility*.” (ATS1).

#### Health

Respondents underscored the relationship between health, racialization and energy use. They recommended health be considered not merely as a dimension requiring energy but also to consider the health outcomes of poor energy use, and how racialized experiences of energy use can contribute to poor health outcomes–"*Again, I'm gonna say health…’cause it's the really obvious one, but maybe they're important things we can do through the energy transition that influence disparities in health outcome by thinking through this energy technology transition pathways*.” (PE1).

#### Food

Respondents stressed the strong cultural ties with food which are important to appreciate with respect to diverse energy practices—for instance, one respondent noted that people who might have to cook food from scratch versus those who might have easier access to ready-to-eat versions of their food, might have different energy use profiles: “*If you're cooking subzi it has to be from scratch, you know, it takes longer. You’re perhaps using more energy, you're using it at a different time of day maybe, you know, which will have an impact on variable tariff situation or the ability to shift demand. So, I mean, if you're making Indian food, it's hot. It's a different fuel base, isn't it?”* (PES1). While food might not drive energy demand to the same extent as buildings or transport, the fuel poverty challenge of “heating or eating” and its strong ties to culture are worth exploring further, as AES1 observes: “*When it comes to climate change, I think food is definitely something that people have a really, you know, rare connection with. And I think also quite often, if you move into another country, I think food becomes even more important in that situation, because it's something that you have from home*.”. Food can also be a way of building trust when engaging with communities, as ATS2 mentioned: “… *basically we used food and cooking as the way to interact with the communities, and we used that to work with them on energy and energy behaviors and use of energy meters and all these other things.*”

### *Processes*

Here we seek to answer the question—how are the energy system’s processes to manage energy use racialized? It is important to observe how behaviours or practices that are encouraged as aspirational to achieving a low carbon lifestyle and the institutional procedures devised to support them might bear a tendency to overlook specific experiences, or in certain cases, perpetuate racialized disadvantages. In this section, we synthesise the participants’ responses around (a) people’s energy use practices, (b) their acceptance and trust of low carbon technologies, (c) their access to energy advice services, and (d) their negotiation of how the energy system is governed, that can contribute to racialized disadvantage.

#### Energy use practices

People have complex and diverse energy practices that may not always lend themselves to obvious energy conservation practices or flexible times of use. As AES11 mentioned: *“It’s really about that agency and scope for people to do things differently. I've seen this in behavioural change research, it is assumed that people are constantly picking from a menu. For a lot of people, for a whole set of reasons, that menu is pretty constrained.*” This also complicates assumptions and definitions surrounding average household energy use. Simultaneously, energy conservation practices of racially disadvantaged people must be recognised as they navigate the energy system, and emulated where appropriate: “*You have situations where a lot can be learned from the experience of different ethnic groups in terms of energy use. Particularly, that it is a more collective endeavour for many groups but I’m going to be careful as we don't want to be sort of romanticising their deprivation…but there are certainly practices around sharing, around collective economies, around collective representations that can be learnt from*.” (AES2) These might include practices such as switching off heating in rooms that are not occupied but we need to be careful that coping mechanisms meant to cope with hardship are not idealised, because— “*Many a times, trauma can resemble culture.”* (PS1).

#### Technology acceptance and trust

There is recognition that different groups engage differently with technologies, depending on how easy they are to use and how intrusive they might appear (for example, the technical possibility of smart meters to convert to prepayment meters). For instance, female household members make the majority of the household choices about energy use, but are not always in charge of purchasing equipment, monitoring energy consumption or paying the bills, especially in the context of increased digitalisation, automation and penetration of smart home technologies. The types of decisions being made in these contexts are different (day-to-day versus one-off decisions) and need to be distinguished from each other, as do the intersections between the different interdependent identities and power relations that people hold in their lives*—“If we are designing and developing stuff and then putting it out in the world, but we have not talked to and included the people who will ultimately benefit or not from this, that is bound to fail. So the classic example is putting heat systems in people’s houses and the control system is built by an engineer or a technical-minded person. It might make sense to them in the lab to know how a thermostat works, but it makes absolutely no sense to the user, who doesn’t really need to know that, but does need to know how to set it to be warmer during the weekend.*” (PES3).

#### Energy advice services

Many racially disadvantaged people, especially recent immigrants and asylum seekers, are unaware of ways to access energy advice services that could support their decisions around energy suppliers and choice of competitive tariffs—for instance, deciding between fixed and standard variable tariffs, and which of those might be most suitable given their energy needs and the suppliers’ responses to changes in wholesale energy prices. In the context of the 2022 energy crisis, AE1 observed that: “*I think minorities tend to be more on the fixed tariff of energy use whereas others are on the variable tariffs and traditionally variable tariffs were cheaper. Uh, and fixed where higher and now if someone is switching to a fixed status, the fixed tariffs are higher and variable tariffs are cheaper, so it will be interesting to see all these differences because there's going to be a lot of movement across these issues as well, just to save up on bills*.” While energy suppliers are obliged to maintain a Priority Services Register (PSR) service of vulnerable customers (including those with extra communication needs or limited knowledge of English), this does not help those who are unaware of it. Language barriers further complicate this— “*If English is not your first language, you cannot effectively obtain the information you want. Your debt repayments might be set too high because it has not been explained clearly to you. An organisation might say you have satisfied the ability-to-pay criteria, but might actually not have had a meaningful conversation in language that is easy for you to understand.*” (PES3). In addition, owing to funding constraints, most of the available advice support is for emergency responses, rather than something more continuous.

#### Governance

The supplier-hub model that has dominated the UK energy system, assumes consumer sovereignty and positions the energy supplier as the primary provider of consumer needs. Their construction of the ‘typical’ energy user tends to overlook the diversity of energy users and their needs, as observed by PE2: “*…like who is the typical user … there's no further understanding of that. It's typically..uh, two ways of thinking about it. One is these so-called sticky customers who don’t switch despite being suggested that they might find a better deal if they shop around, that's how it's been seen historically. Customers don't do that for a variety of reasons. The second is the so-called sort of priority groups or vulnerable groups. And we supply as a priority service register. That's centrally managed and different characteristics are attributed to different households or individuals that then qualifies them for the priority register. And once you are there then suppliers are meant to engage with you differently, and give you direct support in terms of allowing you to be able to pay your bills for a longer period of time, or waive off your debts or something. But there's so much more. There's so many more characteristics of an energy user that are hardly captured basically, and I think that that is another problem.”* Suppliers are obliged to publish quarterly implementation data on their energy efficiency programmes, which includes number of properties insulated, type of insulation, cost, etc., but is silent on the nature of beneficiary households and why they were targeted.

### *Methods*

The practice of research is as important as the research itself. Therefore, this theme engages with the opportunity to practice and sustain anti-racism in energy research. This section explores how to consciously apply principles of anti-racist research methods and attempts to identify the kinds of tools, guides and approaches that can help achieve that.

#### Data collection

Data collection and processing is political, not value neutral. While it is foundational to building evidence, it has its limits. Respondents acknowledged that it is important to be conscious about who is collecting the data, for what purposes, and whose needs are expected to be met by the research: *“…data itself is politicised. This is so at the source itself, if you have inequality, then your entire research is going to be unequal because you're missing out a key variable in terms of looking at that at the source itself*.” (AE1) More systematic, better coordinated research would help to avoid participant research fatigue, and more longitudinal studies would support a more historically-sensitive understanding of the issues, and track their nuances over time. Oversampling the minority groups ensures that the data can be meaningfully analysed, whilst still protecting anonymity—“… *even though ethnicity data is present, because certain ethnicities are a minority in the UK, often there aren't enough participants for them to be meaningfully analyzed. And then you either merge them with another category right? Then you have like you might have Black, dual heritage, Indian, Pakistani, Asian and then one category that's called anything else that you then merge together because like for certain statistical analysis methods, you need to have at least X number from a certain category. And obviously that's something that you might not achieve unless you have oversampled your data to have a lot more. But often the government data is not oversampled. So yeah, I think that's another challenge is small sample size, the category size plus some data sources don't allow separating or using ethnicity to preselect the sample.”* (AES4).

#### People

Researchers should acknowledge their positionality when considering an appropriate research design. In some circumstances, the researchers’ relative positions of power should be responsibly applied to highlight and further particular agendas—making one’s normative position explicit, while acknowledging other approaches, and dimensions (of vulnerability, for example), can be a useful starting point: “*Maybe making your kind of moral, normative starting point clear at the beginning about what your position is, and you know… maybe it's a case of just being open about that upfront somewhere in the introduction and just saying this is the focus, but we still acknowledge other kinds of inequalities as well.*” (AES7) Consideration needs to be given to the framing of participation and its methods – for example, are citizens’ assemblies and participatory budgeting sufficiently representative, and are they appropriate methods to elevate marginalised voices? To be trusted in communities, researchers need to respectfully use community practices and should consider taking bottom-up approaches that work with communities to identify the subject of the research so that it is beneficial to the needs and priorities of the community: “*But you just have to take a lot of time to build trust, to build understanding, to understand where they're coming from. And also, yeah, and at times to deal with people who have very chaotic lives. There does tend to be too much of a tendency among researchers, I think, to think that everyone is fairly on top of things and leads orderly lives, you know?*” (AE3).

#### Conceptual approaches

Social theories rooted in a recognition of intersectionality help make sense of the diversity of people’s experiences of energy use, structural privileges and its consequences on their daily lives: “*… we can pick up from the stats that single parent households headed by females are… disproportionately represented amongst the fuel poor. But do we specifically understand why? And what that means for people's daily lives?”* (AES8). Practicising anti-racist research is an opportunity to further embed social theories within energy research. This helps identify the systemic as well as experiential nature of challenges, consequently informing where best to direct interventions. For example, if income is the predominant challenge, responses could focus on institutional schemes to address that, though mistrust in formal institutions or being ineligible for state support might mean such ‘solutions’ need to be delivered differently, and traditional ‘units’ of analysis such as households and ‘families’ rethought: “*In our discourse analysis of energy poverty policies across Europe, we find that they're framed in a very narrow way, and we find that they're quite ideologically driven. So, for example, Catholic countries talk about how they can support large families through difficult, you know, energy crises, *etc*. But actually, we know from the stats that single parent households headed by females are much more vulnerable to energy poverty across Europe. So, there's this disjuncture between evidence and ideology. And so, I think that method [discourse analysis] has been so very revealing.*” (AES8).

#### Analysis

Like data collection, analysis is also not value neutral, as previously observed by AE1. Respondents recognised that the causal explanatory power of most quantitative studies remains limited, and suggested intervention-oriented approaches such as realist evaluations or social-impact assessments to provide useful contributions to this limitation: “*So it's recognizing what it is actually, you know, what is actually happening, what is the mechanism that's leading to that experience and where can that be intervened upon, at the kind of structural level. And then you can use that to help design your intervention and then look for evidence or as to whether or not that's been effective or whether it's something you might test out.*” (AE4) Qualitative approaches such as discourse analysis, as highlighted by AES8’s quote above, were found to be informative in explaining the disjuncture between evidence and ideology. Respondents also recognised that it is the fundamental nature of some evidence to be political. For example, good air quality and access to green spaces are correlated with better health, and there is ample evidence that access to these is racialized. The implication is that by depriving some parts of the community of green spaces, their well-being may be compromised, and this responsibility of appropriately interpreting the analysis lies with the researcher.

## Discussion

### *Contextualising the findings in the literature*

This research began by exploring the reasons for the persisting dearth of research that explores energy use and racialization. The findings demonstrated that one of the challenges concerns linking contemporary everyday experiences of energy use with systemic (and historic) injustices. One of the ways that this manifests in existing literature is through the tendency to conflate class and race, with unhelpful hierarchies being created between representation versus redistribution that risks overlooking experiences (Fraser, 1998)—for example, whilst the UK’s leadership in fuel poverty research is widely recognised (Bednar & Reames, 2020), it rarely includes an analysis of racialization as a contributing factor even though it is acknowledged that racially disadvantaged people are overexposed to fuel poverty risks (Blakelock, 2021; Bouzarovski et al., 2022; Middlemiss, 2022). As noted by Blakelock (2021), data pertaining to race and ethnicity characteristics (which is what most data on racialization is usually reduced to) is not collected often, nor across all relevant datasets.

The findings identified three ways to address these challenges. The first is concerned with the need to strengthen evidence pertaining to the energy services that might be most susceptible to racialized disadvantages. The built environment not only determines people’s energy use but also their access to technologies such as solar PV, heat pumps or smart meters, that can be challenging to install in multi-storeyed housing projects, where racially disadvantaged people are overrepresented (Danewid, 2022; Gulliver, 2017), thereby providing a more comprehensive appreciation of the spaces used by people. For transport, another significant driver of energy demand, apart from corroborating Gates’ (2009) observations pertaining to how access to transport services contributes to spatial inequalities, and the complexities surrounding air travel (Mattioli & Scheiner, 2022), the findings also discovered the racialization of precarious mobile work that warrants further investigation. Health has a dual relationship with energy use at it influences and can be influenced by energy use and systemic inequalities (Camargo, 2020), and healthcare workers could also act as crucial intermediaries in research involving community engagement (Creutzfeldt & Gill, 2021). And while food is not a significant contributor to energy demand, it is an energy service that warrants further study especially to understand its energy requirements, as well as the role it plays in building community and a sense of belonging.

The second approach to address the dearth in research engaged with understanding the energy system’s processes to manage energy use. When discussing energy use practices, respondents corroborated the observation by Hodges et al. (2022) that energy users from multi-generational households (largely represented by people from an Asian background (ONS, 2022) possessed energy use profiles that could not always be as flexible. While research recognises the role of public acceptance of decarbonisation technologies, there is not enough work on who constitutes that ‘public’. The findings underscored the recognition that energy research’s understanding around the experiences of racially disadvantaged people’s acceptance, access to and use of decarbonising technologies is nascent. With respect to accessing timely energy advice, the responses corroborated that people who have experienced discrimination from authorities also feel uncomfortable accessing services, even those they are entitled to receive (Hodges et al., 2022).

And finally, the methods to address the research dearth require moving beyond essentialising categorisations like ‘ethnic minority’. Properly informed research should ask specific questions, understanding how issues affect different people, so as to recognise the participants’ experiences and not essentialise them within homogenising categories. Harmonising this data between the four nations of the UK will also help (as ‘ethnic minority’ is interpreted differently across them (GOV.UK, 2011; GOV.UK, n.d.). Case studies should exemplify a range of experiences rather than selecting reified cases because they might be convenient. It is well acknowledged in energy research that consumers’ levels of agency to make ‘rational’ choices vary. For instance, where one lives, in what kind of house, significantly influences heating and transport ‘choices’. Research about energy user experiences (especially around energy source), type of energy supplier (decentralised, market-oriented), and type of energy user are relevant in this context. Understanding users’ experiences is crucial to the design of interventions and to inform policy. Linking this experiential data with an understanding of how institutional mechanisms perpetuate exclusion ensures that racially disadvantaged people are not blamed for practices that actually emanate from such structures. In some cases, the work may be extremely sensitive, such as with groups exposed to higher criminalisation. Where necessary, engaging through trusted intermediaries, such as NHS case workers or community leaders, is a useful way of engaging with them. When doing this work, it is essential to compensate people for their time and contribution, acknowledge their involvement by co-producing the research outputs, and actively challenge traditional boundaries of the researcher and the ‘researched’ and the power dynamics of those relationships.

### *Avenues for future research: themes*

**Fig. 3:**
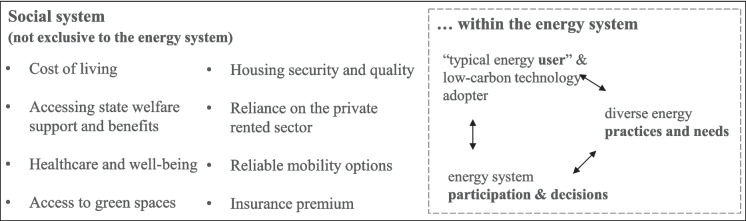
The energy system as a ‘racialized organisation’ within a racialized social system, and avenues for future research

Investigating the racialization of the energy system requires understanding racial disadvantage at a societal level, and exploring how this might manifest within the energy system (Fig. 3). Therefore, we propose the following questions to initiate further conversations and commitment towards anti-racist energy research:

- Who is assumed to be a typical energy user or early adopter of technologies that will aid in rapid decarbonisation? What does that mean for those who might not fit that assumption in terms of accessing the services and technologies to assist low-carbon practices? As Newell (2021) observes, “the socially uneven impacts of energy transition pathways” need to be investigated. The graphical abstract to Middlemiss’ (2022) paper is an artwork by Mary Tallontire, depicting a Black single mother huddling with her children under a blanket to watch something on a laptop—demonstrating the intersecting identities and experiences of those in energy poverty who risk being left behind in the transitions taking place in the energy system. Research from the United States demonstrates how some of the practices structurally embed inequalities, such as the lower availability of energy efficient lights in racially disadvantages neighbourhoods of Detroit (Reames, 2016; Reames et al., 2018).
- Do we understand the diverse and complex energy needs and practices of racially disadvantaged people? How responsive or inelastic could those practices be to what is considered popularly as low carbon lifestyles? In a study conducted in Bradford in northern England, Owen et al (2023) discovered that low-income, owner occupied households of Asian origin, living in dwellings with poor energy efficiency are significantly more likely to apply for government schemes to improve energy efficiency. These findings challenge a long-standing assumption that households belonging to such demographics are ‘hard-to-reach’ (as an exception, refer to Rotmann, et al. (2020) for a comprehensive literature review on hard-to-reach energy users).
- Are existing opportunities to participate in the energy system racialized, thereby contributing to unfair decisions? Could that affect the way problems are defined and solutions are designed?

Additionally, there are two emerging themes around energy system participation that this conceptualisation of racialization and energy can potentially contribute to—'energy communities’ and ‘energy citizenship and democracy’. As Silvast and Valkenburg (2023) observe, existing literature on who is considered an energy citizen needs to be addressed more explicitly and its relation to more traditional understandings of citizenship (which are bordered and racialized, as El-Enany (2020) demonstrates in the case of Britain, for instance) is less clear. They also note that while literature tends to assume that energy citizenship and energy democracy develop in tandem, that is not always the case. Especially as demonstrated by growing evidence around renewable energy communities (RECs) and who participates in them. With RECs gaining wider acceptance and becoming mainstream in policy considerations, especially in Europe, considerations around the level of inclusivity and fairness within these communities is becoming imminent. For example, Hanke and Lowitzsch’s (2020) research exhorts for RECs to enable vulnerable energy consumers to participate in them, recent work by Ettwein and Werner (2023) demonstrates that energy communities are not inherently fair and draw our attention towards challenges of injustice that energy communities might be prone to, in their formative stages. Most of this research has focused on the classed and/or gendered nature of RECs, thus providing an opportunity for future research and action to ensure RECs acknowledge the relative power held by their participants while attempting to become more inclusive. Strengthening evidence on the risk of racialization within energy system participation, especially by exploring the findings pertaining to energy services and processes can be a useful starting point.

### *Avenues for future research: approaches and methods*

Racialized disadvantages persist because of their ability to constantly redefine ‘the other’ (Meer, 2022; Meghji, 2020; Glynn, n.d.). Therefore, as actors engaging with work on racial justice, it is important to resist essentialising labels such as ‘BAME’ and ‘race’ and adopt more flexible ways of understanding and articulating the processes of racialization, and how it impacts identity. Such research is often caught in an analytical bind between the risk of homogenising diverse experiences or being entrapped by identity politics (Fraser, 1998). A transversal approach could provide a useful starting point to respond to this (Anthias, 1998; Cannon & Chu, 2021; Yuval-Davis, 1999, 2006, 2017). It is helpful to reflect on how we might minimise the risk of homogenising people’s experiences by being sensitive to inter- and intra- group dynamics around power and privilege. A transversal approach is also useful for interpreting intersectionality beyond identity by also considering positions and values. This approach is based on three principles (Yuval-Davis, 1999). Firstly, it recognises differences in standpoints (and does not privilege one over another, beforehand) and believes that a dialogue between these different positions is an important practice. The second principle is difference by equality which means that “notions of difference should encompass rather than replace notions of equality” (Yuval-Davis, 1999: 95). It also acknowledges that people who identify with a social group identity might be positioned differently across other divisions (such as class, gender, age, etc.) and hold very different values, socially and politically. This observation helps us to understand the different ways in which social divisions are constructed by, and intermeshed with each other, in specific historical conditions; thereby providing an nuanced understanding of power dynamics. Such an understanding can help challenge reductive binaries like ‘engaged versus hard to reach’ or ‘active/prosuming versus passive’ energy users, that risk perpetuating energy injustices towards already marginalised people.

**Fig. 4:**
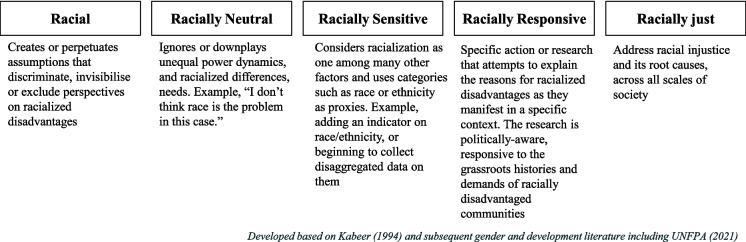
The racial justice continuum (Narayan, 2023)

Inspired by action-oriented contributions from gender and development, we propose a racial justice ‘continuum’ (Fig. 4) to articulate anti-racist ambition in conducting energy research (Kabeer, 1994; Ryan, 2014; UNFPA, 2021). The continuum consists broadly of five types of research, arranged in order of least aspirational (i.e. racial research) to most aspirational (i.e. responsive and racially just research). Racial research is based on assumptions that discriminate, invisibilise or exclude perspectives on racialized disadvantages, intentionally or otherwise. Racially neutral research ignores or downplays unequal power dynamics and racialized differences and needs. Racially sensitive research considers racialization among other factors, but tends to use static proxies such as race and ethnicity categories. Racially responsive research can be specific and context-sensitive, that attempts to explore the explanatory factors contributing to racialized disadvantage. It will also be reflective of the power dynamics involved in the performance of research, and applies principles of transversality, not just to the subjects of research, but also to the relationship between the researchers and the researched. Devine-Wright and Ryder (2024) provide some specific recommendations on ‘place-based reflexivity’ to address the asymmetry of power and risk of extractive practices in energy social sciences research. Finally, racially just research would systemically transform the research landscape as it tackles the root causes behind racialized disadvantages. This is framed as a continuum since the boundaries between some of these categories of research might not be definite. However, these categories of research need not be interpreted sequentially. What this means is that, for a racially neutral piece of research to become racially responsive, it is not required for it to first be sensitive and then become responsive. The objective of presenting this as a continuum is to demonstrate that research that aspires to be racially justice, exists across a spectrum.

While we acknowledge that research alone cannot deliver racial justice, a commitment towards ensuring that energy research qualifies for the criteria outlined under the ‘responsive’ category, is a reasonable ambition to hold. While applying this continuum, it is important to be conscious that the framing is not a teleological vision of achieving racial justice (Seamster & Ray, 2018), and must be interpreted appropriately, as a suggestive scaffolding to think more proactively about anti-racist energy research.

## Conclusion

This research was envisaged to begin understanding ways to conduct anti-racist research on energy use, however, it is not devoid of limitations. Though there is some evidence on the nature of racialized disadvantages experienced by different social groups, their interactions with other actors in the energy system requires further investigation. This scoping research is limited to the UK, and the racialization of the energy system might manifest differently in another context and scale. This research also acknowledges the limitations to being able to conduct anti-racist research—ranging from structural (such as participating in the energy system that might be dependent on controversial energy infrastructures, and the positionality of researchers) to semantic (negotiating the use of terms like BAME or race that are recognised as problematic (British Future, 2021; Mistlin, 2021), but most publicly available data is only collected along these categories). It is important to acknowledge and accept that research of this nature is political, and can be uncomfortable (Pulido, 1996). It is important to hold safe spaces, but also engage with the discomfort as part of dismantling systems of injustice, and offering “care across differences” (Eaves et al., 2023). However, this should not compel us to force-fit a singular definition of what anti-racism ‘should be’ (Pulido, 1996). Racialization and the creation of racial meaning is an on-going process. Therefore, the objective of this effort is to create a pluralistic analytical space, that exists amidst the contradictions and multitudes of social relations with and within the energy system (Bouzarovski, 2022; Hargreaves & Middlemiss, 2020; Sovacool et al., 2023).

By drawing on the tradition of Critical Race Theory, and specifically, the racialized social system approach, this paper attempts to provide a conceptual starting point for thinking about energy use and racialization in a critically robust manner. As demonstrated in the reasons for the dearth of such research, the findings discovered that systemic racialization within the energy system might not appear obviously associated with everyday experiences of accessing energy services. This is one of the conceptual contributions of Bonilla-Silva’s (2021) racialized social system approach in highlighting that racialization is systemic and not limited to interpersonal acts that might be evaluated as prejudice. Anchoring this research in the concept of the racialized social system approach has allowed us to not run the risk of thinking about the energy system in isolation from other social and socio-technical systems. Recognising the energy system as a meso-level organisation in the racialized social system allows us to understand the ways in which it influences and is influenced by social structures, mechanisms and relations. This also helps to think more expansively about energy justice. Energy justice is most commonly conceptualised at a systemic level through three tenets—namely, distributional, procedural and recognition, and distinguishes itself from more grounded, activism-inspired environmental and climate justice movements, as a top-down approach (Jenkins, 2018). Damgaard et al. (2022) partially address this dissonance through the contribution at the interpersonal level by introducing care ethics to energy justice considerations. While these theorisations take into consideration the systemic and the interpersonal, the racialized social system approach contributes to the meso-level, thereby also attempting to bridge agency and structure, within theorisations around energy justice. Here, it is also important to acknowledge Wood’s (2023) critique of the three tenet approach and ensure that anti-racist energy research acknowledges its political origins in social movements through proactive engagement with the grassroots, and does not get appopriated as an ahistoric, technocratic instrument (Pulido, 1996). Such a conceptualisation also allows us to think of the scales across which responsibility and action towards racial justice can be considered, as attempted by the racial justice continuum (Fig. 4).

The following quote from an interview respondent summarises the spirit of this research—*"In the crisis that we have in the energy market at the moment, it is very difficult for me to see how we will ever build a system which will get us to net zero without understanding the different ways in which people use energy and interact with the machines that they've got in their homes and in their system. If we don't understand that, then we're just not going to get to net zero at all. But we've got this kind of double work to do. The actual work itself to understand, but then also the work of convincing people that it's worth understanding.”* (PES2).

We acknowledge that the sources of these perspectives are researchers and practitioners in the energy system. They might be considered as more privileged actors in relation to those who experience racialized disadvantages in using energy—in future participatory research with a neighbourhood community, we aim to address this limitation and apply lessons from this scoping research to understand how communities feel racially disadvantaged in using energy and what their expectations are from systemic actors, who bear the responsibility to ensure that their energy needs are met. While this can be considered a next step, this paper seeks to shine a light on the type of research required to strengthen our understanding of the relationship between racialization and energy use, as well as be conscious of the ways in which we conduct such research, as research of this nature can be vulnerable to being unequal and extractive. This understanding is imperative to transition to an energy system that is not only clean but also equitable, especially in terms of how people can participate in such a system.

## Supplementary Information

Below is the link to the electronic supplementary material.

Supplementary file1 (PDF 56.2 KB)

## Footnotes

1 These are respondent signifiers. Semi-structured interviews were conducted with academic researchers and practitioners who identified as experts on energy and/or social research in the UK. AE: Academic (Energy), AES: Academic (Energy & Social Research), ATS: Academic (Transport & Social Research), PE: Practitioner (Energy), PS: Practitioner (Social Research), PES: Practitioner (Energy & Social Research). The numbers merely signify the sequence in which they were interviewed. All the quotes are from the semi-structured interviews.

## Declarations

### Availability of supporting data

Consent was not sought to publish anonymised transcripts and therefore is not available. However, the coding scheme for the qualitative analysis and summary of existing literature on racialization and energy demand is available as supplementary material to this paper.
