# Supplementary material for: Approaches to address the gap in research on energy use and racialization in the UK
Source: Energy Effic. 2026 Jan 8;19(1):4. doi: 10.1007/s12053-025-10390-6 (PMC12783292; doi:10.1007/s12053-025-10390-6)
Supplement: Supplementary file 1 — Supplementary file1 (PDF 56.2 KB) [file 12053_2025_10390_MOESM1_ESM.pdf]

SUPPLEMENTARY MATERIAL

1. Summary of existing literature on racialisation and energy use

The first round of review started with a simple search on ScienceDirect database using "race" and "energy demand" as the search terms. Of the results that showed up 22 papers were filtered for the first round of review, given their explicit consideration of race and ethnic minorities in their publication, rather than as a broader normative statement around energy justice (for e.g. publications that mention and acknowledge that racial justice is important, but might not actively address that in the same publication, as the objectives might be different). Other papers were identified through snowballing, based on their citation in these papers. A total of 28 papers were reviewed in this round. The second round involved applying a combination of (energy, energy demand, energy poverty, energy justice, fuel poverty) and (racial justice, racialisation, race, ethnicity) terms to the International Bibliography of the Social Sciences (IBSS) database, that yielded an additional 7 papers to the review, taking the total of the review to 34 publications. Additionally, two more publications were added to the review, as they were recently published articles that were publicised through research lists and mentioned in the interviews by experts, and some grey literature such as think tank reports, legal cases and newspaper reports were identified. Subsequently, it was discovered that Energy Economics ran a journal special issue on race and energy, thus taking the final list of publications to 59. The following table enlists all the identified literature:

Table i: List of publications identified in the scoping literature review and presented in Figure 2 in the paper

Citation	Year	Demand / Supply focus	Sectors	Analysis Type	Data Source	Geographic coverage	Analysis level
**Poyer & Williams 1993**	1993	Demand	Residential	Quantitative	Secondary	US	National
**Poyer et al 1997**	1997	Demand	Residential	Quantitative	Secondary	US	National
**Newell 2005**	2005	Both	Energy System	NA	NA	Global	Global
**Adua & Sharp 2011**	2011	Demand	Residential	Quantitative	Secondary	US	National
**Reames 2016**	2016	Demand	Residential	Qualitative	Primary	US	City
**Reames 2016**	2016	Demand	Residential	Quantitative	Secondary	US	City
**Bednar, Reames and Keoleian 2017**	2017	Demand	Residential	Quantitative	Secondary	US	City
**Lennon 2017**	2017	Supply	Energy System	Qualitative	Primary	US	City
**Churchill & Smyth 2017**	2017	Demand	Residential	Quantitative	Secondary	Global	National
**Reames, Reiner and Stacey 2018**	2018	Demand	Residential	Quantitative	Primary	US	County
**Valencia 2018**	2018	Supply	Energy System	Qualitative	Primary	Venezuela	Region
**Forster, Hodgson & Bailey 2019**	2019	Demand	Residential	Qualitative	Primary	UK	Region
**Sunter, Castellanos & Kammen**	2019	Supply	Residential	Quantitative	Secondary	US	National

**Bednar & Reames 2020**	2020	Demand	Residential	NA	NA	US	National
**Kontokosta, Reina and Bonczak 2020**	2020	Demand	Residential	Quantitative	Secondary	US	City
**Lewis, Hernández and Geronimus 2020**	2020	Demand	Residential	NA	Primary	US	National
**Lyubich 2020**	2020	Demand	Residential	Quantitative	Secondary	US	National
**Mhlanga and Garidzirai 2020**	2020	Demand	Residential	Quantitative	Secondary	South Africa	National
**Nature 2020**	2020	Both	Energy System	NA	NA	US	National
**Reames 2020**	2020	Supply	Residential	Quantitative	Secondary	US	City
**Churchill & Smyth 2020**	2020	Demand	Residential	Quantitative	Secondary	Australia	National
**Blakelock 2021**	2021	Demand	Residential	NA	NA	UK	National
**Graff et al 2021**	2021	Demand	Residential	Quantitative	Primary	US	State
**Lennon 2021**	2021	Both	Energy System	NA	NA	US	City
**Newell 2021**	2021	Both	Energy System	NA	NA	Global	Global
**Phillips & Petrova 2021**	2021	Both	Energy System	Qualitative	Primary	South Africa	City
**Reames 2021**	2021	Supply	Residential	Quantitative	Secondary	US	National
**Turner 2021**	2021	Demand	Residential	Quantitative	Secondary	UK	National
**Wang et al 2021**	2021	Demand	Residential	Quantitative	Secondary	US	National
**Jayasinghe et al 2021**	2021	Demand	Residential	Quantitative	Secondary	Sri Lanka	National
**Paudel 2021**	2021	Demand	Residential	Quantitative	Secondary	Nepal	National
**Sedai et al 2021**	2021	Supply	Residential	Quantitative	Secondary	India	National
**Bouzarovski, Burbidge et al 2022**	2022	Demand	Residential	Mixed Methods	Both	UK	National
**Goldstein, Reames & Newell 2022**	2022	Demand	Residential	Quantitative	Secondary	US	National
**Chen et al 2022**	2022	Demand	Residential	Quantitative	Secondary	US	County
**Chen, Greig et al 2022**	2022	Demand	Residential	Quantitative	Primary	US	State
**Dogan et al 2022**	2022	Demand	Residential	Quantitative	Secondary	US	National

**Sovacool & Rio**	2022	Demand	Energy & Transport	Qualitative	Primary	UK	National
**Hodges, Schmidt & Becker**	2022	Demand	Residential	Qualitative	Primary	UK	City
**Saunderson**	2022	Demand	Residential	NA	Primary	UK	City
**Middlemiss**	2022	Demand	Residential	NA	NA	UK	Region
**Mattioli & Scheiner**	2022	Demand	Transport	Quantitative	Secondary	UK	National
**Ngarava et al 2022**	2022	Both	Residential	Quantitative	Secondary	South Africa	National
**Leslie et al 2022**	2022	Demand	Residential	Quantitative	Secondary	Australia	State
**Paudel 2022**	2022	Demand	Residential	Quantitative	Secondary	US	State
**Maruejols 2022**	2022	Demand	Residential	Quantitative	Secondary	Vietnam	National
**Chapman et al 2022**	2022	Both	Energy System	Quantitative	Primary	US	National
**Koomson et al 2022**	2022	Demand	Residential	Quantitative	Secondary	South Africa	National
**Koomson & Churchill 2022**	2022	Demand	Residential	Quantitative	Secondary	South Africa	National
**Sovacool et al 2023**	2023	Both	Energy System	NA	NA	Global (but largely US centric epistemology)	Global
**Owen et al 2023**	2023	Demand	Residential	Quantitative	Secondary	UK	City
**Open Society Justice Initiative**	n.d.	Demand	Residential	Qualitative	NA	Bulgaria	National
**Alkhalili et al 2023**	2023	Supply	Energy System	Qualitative	Primary	North Africa	Region
**Baik et al 2023**	2023	Demand	Residential	Quantitative	Secondary	US	National
**Jacobsen 2024**	2024	Demand	Residential	Quantitative	Secondary	US	National
**Nepal et al 2023**	2023	Supply	Residential	Quantitative	Primary	Nepal	National
**Best 2023**	2023	Supply	Residential	Quantitative	Primary	Australia	National
**Lin & Okyere 2023**	2023	Demand	Residential	Quantitative	Secondary	South Africa	National
**Crago et al 2023**	2023	Both	Residential	Quantitative	Secondary	US	State

2. Coding scheme

Level	Code Name	Description	References
1.	Emerging enquiries	This code will capture reflections by respondents on what they think is either (a) under-researched or (b) where new perspectives of research can be contributed to by the proposed research agenda.	
1.1.	Evidence (*methods*)	Sub-code under Emerging enquiries. This will capture the areas where the respondent reflects that sufficient evidence currently does not exist and hence will need to be generated either to strengthen the hypothesis, or to decide on whether the evidence is sufficient to proceed with the current line of enquiry	(Blakelock 2021)
1.1.1.	Built environment (*theme/ outcome*)	Sub-code under Evidence. The term 'built environment' is deliberately used instead of housing, because of the explicit recommendation by interview respondents to consider the built environment more comprehensively rather than look at housing (and household), as a limited unit of analysis. This code captures all the emerging enquiries/ areas requiring further evidence	(Zewolde, et al. 2020), (Huebner, et al. 2022)<br>Housing (Gulliver 2017), (Willis 2018), (D'Souza and Khan 2021), (Raslan and Ambrose 2022), (Danewid 2022)<br>Planning (Rutten 2020)<br>Green space (The Ramblers 2020)
1.1.1.1.	Private Rented Sector (*theme/ outcome*)	Sub-code under Built environment. This covers the aspects of the private rented sector that are relevant to the racial justice research.	(Bouzarovski, Burbidge, et al. 2022)
1.1.2.	Health (*theme/ outcome*)	Sub-code under Evidence. This code captures the various dimensions related to health that were brought up across the various interviews, and how health is one of the primary themes where racialisation is observed, its relationship with other energy uses and outcomes of accessing energy services, and can be used as an entry point to strengthen conversations around race.	Energy insecurity and health: (Hernandez 2016)<br>Race and health: (Camargo 2020), (Race Equality Foundation n.d.)<br>Built environment and subjective wellbeing (Huebner, et al. 2022)
1.1.3.	Intermediaries (*People/ engagement*)	Sub-code under Evidence. This links to engagements but also considers studying the best way to engage with various stakeholders.	(Creutzfeldt and Gill 2021), (Hodges, Schmidt and Becker 2022)
1.1.4.	Intersectionality (*methods*)	Sub-code under Evidence. Code to capture multiple dimensions and intersecting identities of energy research.	(Yuval-Davis 2006)

Level	**Code Name**	**Description**	**References**
			Energy specific (Ryan 2014), (Cannon and Chu 2021); older and multi-generational households (Hodges, Schmidt and Becker 2022) transport and links to migration, ethnicity and geography (Mattioli and Scheiner 2022)
1.1.5.	Net Zero (*theme/ outcome*)	Sub-code under Evidence. This code will capture information on possible avenues of research that is required to make net zero more equitable. This could also be linked to demand-side infrastructure such as new low-carbon technologies to aid in demand reduction/ achieving net zero	(Newell 2021), (Middlemiss 2022), (Raslan and Ambrose 2022)
1.1.6.	Practices & cultures (*theme/ outcome*)	Sub-code under Evidence. This code captures information on the need for further information on how racialised peoples' practices and how that may influence energy demand. This also then links to currently available policy solutions--especially around flexibility of use, where some practices might be inelastic to such use for various reasons and need to be understood better.	(Hodges, Schmidt and Becker 2022)
1.1.6.1.	Food (*theme/ outcome*)	Sub-code under Evidence. This code captures dimensions of food cultures that inform energy demand, and where further investigation might be required.	(Martiskainen, et al. 2021), (Reames, Reiner and Stacey 2018), (T. G. Reames 2016)
1.1.7.	Procedural, regulatory aspects (*theme/ outcome*)	Sub-code under Evidence. This code captures the regulatory and procedural aspects that might contribute to further discrimination or aim to address them, and the evidence required for that.	(Newell 2021), (Creutzfeldt and Gill 2021), (Hodges, Schmidt and Becker 2022), (Citizen's Advice n.d.), (Bouzarovski, Burbidge, et al. 2022), (Forster, Hodgson and Bailey 2019), Bills and expenditure: (Mahon 2022),
1.1.8.	Technology (*theme/ outcome*)	Sub-code under Evidence that looks at how people access and use technologies that are expected to contribute to the energy transition	(Lennon 2017)
1.1.8.1.	Community Local Energy (*theme/ outcome*)	Sub-code under Technology. This code attempts to understand the potential of local energy communities in being inclusive and fair.	(T. G. Reames 2016)

Level	Code Name	Description	References
1.1.8.2.	Cooling-Heating (*theme/ outcome*)	Sub-code under Technology. Several respondents mentioned adding cooling as a significant component to explore further, also because heat vulnerability in the UK in highly racialised.	(Kidwell & Ogunbode, 2022)
1.1.8.3	EVs (*theme/ outcome*)	Sub-code under technology to capture the necessary evidence that needs to be built with respect to the popularity and propagation of electric vehicles	
1.1.9.	Transport (*theme/ outcome*)	Sub-code under Building evidence. Transport is a significant energy use that is also racialised. This code captures information on areas within transport where further evidence needs to be built.	(Schwanen 2018), (Mattioli and Scheiner 2022), (Gates, et al. 2019)
1.2.	Comparative (*methods*)	Sub-code under Emerging enquiries. This is conduct research in the UK context, building on established results or theories of change from other contexts. Also includes observations on what we know already.	(Cannon and Chu 2021)
1.3.	Critical enquiry (*methods*)	Sub-code under Emerging enquiries. This will capture the areas where the respondent reflects that an overhaul in existing systems of thinking is required, or the dominant narratives need to be challenged	(Bouzarovski 2022)
1.3.1.	Racial Justice (*theme/ outcome*)	Sub-code under Critical enquiry. How do we envision racial justice, and how can that vision conceptually contribute to energy demand research?	(Meer 2022), (Seamster and Ray 2018), (Modood 2016), (Kothari 2006), (Phillips and Petrova 2021), (Allas, et al. 2020), (Taiwo 2022)
1.3.1.1.	Colonialism, Racial Capitalism	Sub-code under Racial Justice. This code captures articulations of racial justice that are based on dimensions of colonialism, racial capitalism, historic injustices.	(Bhambra and Newell 2022), (Danewid 2022), (Lennon 2021)
1.3.1.2.	Energy Justice Tenets (*theme/ outcome*)	Sub-code under Racial Justice. This code captures articulations of racial justice by respondents that uses the principles most commonly found in energy justice literature.	(Jenkins, et al. 2020)
1.3.1.3.	Opportunities to access services (*theme/ outcome*)	Sub-code under Racial Justice. This captures what is envisioned as achieving racial justice through material dimensions	(Creutzfeldt and Gill 2021), (Bouzarovski, Burbidge, et al. 2022), Affordability/ advice (Forster, Hodgson and Bailey 2019)
1.4.	Focus—Geographies (*people/ engagement*)	Sub-code under Emerging enquiries to identify the geographies that need to be prioritised. Also, reflections on the spatial perspectives of this research agenda.	(Bouzarovski and Simcock 2017), (Lewis, Hernández and Geronimus 2020)

Level	**Code Name**	**Description**	**References**
			Northern Ireland (Sovacool and Rio 2022)
1.5.	Focus—Groups (*People/ engagement*)	Sub-code under Emerging enquiries to understand the groups that need to be prioritised in taking this research forward	Travellers: (Sovacool and Rio 2022), (Forster, Hodgson and Bailey 2019)<br>South Asian: (Hodges, Schmidt and Becker 2022), (UKERC 2022)
1.6.	Methods & Approaches (*methods*)	Sub-code under Emerging enquiries. This code collects approaches and tactics suggested by respondents on either (a) how they would do things differently on previous projects, or (b) how we can aim to be successful with new research we expect to build	
1.6.1.	Awareness & political education (*methods*)	Sub-code under Methods & Approaches. This code captures the responses where improving awareness and creating (political) education are identified as tactics to mainstream racial justice in the research, and better understand the role of relevance of race in this research.	(Kapoor, Hood and Youssef 2022)
1.6.2.	Data collection (*methods*)	Sub-code under Methods & Approaches. To monitor/ track/ evaluate data to inform more diversity and inclusion	(Ahmadzadeh 2021)
1.6.2.1.	Disaggregation (*methods*)	Sub-code under data collection. Lack of disaggregated data especially around ethnicity has been identified in literature and vocalised by respondents in terms of a critical research gap that needs to be addressed	(Yuval-Davis 2006), (Bouzarovski, Burbidge, et al. 2022)
1.6.2.2.	Evaluation (*methods*)	Sub-code under Data collection. This code looks at examples or ways of monitoring and evaluation that is important to further evidence on race and energy use	(Fortier, et al. 2019), (Jenkins, et al. 2020), (Gates, et al. 2019)
1.6.2.3.	Historic contextualisation (*methods*)	Sub-code under data collection. This code captures reflections on historic developments (and will be related to inputs from respondents on key moments in British history), to ensure that this research does not fall into a teleological methodology gap, but is grounded in historic material developments.	Teleology of race: (Seamster and Ray 2018)
1.6.2.4.	Qualitative (*methods*)	Sub-code under Building evidence. This code captures how respondents reflect on capturing lived experiences and related methods to strengthen the research agenda.	Lived experience (Middlemiss 2022), Mixed methods (Bouzarovski, Burbidge, et al. 2022)

Level	Code Name	Description	References
1.6.2.5.	Quantitative (*methods*)	Sub-code under Building evidence. This code captures how respondents reflect on capturing quantitative methods to strengthen the research agenda.	Spatial modelling: (Bednar, Reames and Keoleian 2017) Modelling justice: (Menghwani, et al. 2020)
1.6.2.6.	Spatial (*methods*)	Sub-code under Data collection. This code captures spatial and geographic dimensions of the emerging research	(Bouzarovski and Simcock 2017), (Lewis, Hernández and Geronimus 2020), (Neely and Samura 2011), Spatial modelling: (Bednar, Reames and Keoleian 2017)
1.6.3.	Engagement (*People/ engagement*)	Sub-code under Methods & Approaches. Ways of engaging with various stakeholders on this research, especially in terms of tactics to build trust, and make the process inclusive and participatory.	(Creutzfeldt and Gill 2021), (Hodges, Schmidt and Becker 2022), (UKERC 2022)
1.6.3.1.	Stakeholders (*People/ engagement*)	Sub-code under Engagement. This code will collect the references all types of stakeholders mentioned in the consultations	
1.6.4.	Funding (*methods*)	Sub-code under Methods & Approaches. Types and sources of funding that can be considered to strengthen the evidence/ research base.	(Blakelock 2021)
1.6.5.	Language (*methods*)	Sub-code under Methods & Approaches. This code captures information around the need to be deliberate and conscious about the language we use to address the challenges, and communicate effectively how to address them.	(Ahmadzadeh 2021), (British Future 2021), (Mistlin 2021)
1.6.6.	Policies (*methods*)	Sub-code under Methods & Approaches. Code will capture policy and formal institutional mechanisms suggested by respondents to further the agenda.	(Bednar and Reames 2020), (Raslan and Ambrose 2022), (UKERC 2022)
1.6.7.	Representation (*People/ engagement*)	Sub-code under Methods & Approaches. To capture who mentions diversity and how can diversity be improved, and does greater diversity lead to better outcomes? It is also important to recognise (and problematise) tokenism in the name of representation and truly ensure that representation means tackling unequal power distribution	(Blakelock 2021), (UKERC 2022), Kapadia (2021)

Level	Code Name	Description	References
2.	Gaps	What are the existing gaps in energy demand research that have resulted in not looking at race and racial justice as an explicit dimension? Why do these gaps persist?	
2.1.	Data (*methods*)	Sub-code under Gaps. Lack of sufficient data to support the need for an explicit consideration of racial justice. Also, current data infrastructure is insufficient to capture the complexities of understanding race relations in the UK, and therefore envisioning racial justice.	(Fortier, et al. 2019), (Jenkins, et al. 2020), (Gates, et al. 2019)
2.1.1.	Availability (*methods*)	Sub-code under Data. Is there sufficient data? Is it comprehensively collected? Is it sufficiently representative? How easily is it available/ accessible?	Teleology of race: (Seamster and Ray 2018)
2.1.2	Limited or poor classification of data (*methods*)	Sub-code under Data. Data infrastructure. Is the data not sufficiently self-explanatory? Is it buried under other attributes, that makes an explicit analysis difficult? Or, as current articulations of data insufficient to understand the complexities?	Lived experience (Middlemiss 2022), Mixed methods (Bouzarovski, Burbidge, et al. 2022)
2.1.3.	Unequal distribution of data (*methods*)	Sub-code under Data. Is there an implicit bias in the way existing data is structured that leads to a structural challenge in capturing this information? The unequal distribution of data can be geographically or nature of data-wise (quantitative versus qualitative data)	Spatial modelling: (Bednar, Reames and Keoleian 2017) Modelling justice: (Menghwani, et al. 2020)
2.1.3.2.1.	Sectors (*theme/ outcome*)	Sub-code under Unequal distribution of data. This identifies under-researched areas/ sectors where more explicit focus might be required.	(Bouzarovski and Simcock 2017), (Lewis, Hernández and Geronimus 2020), (Neely and Samura 2011); Spatial modelling: (Bednar, Reames and Keoleian 2017)
2.2.	Material (*theme/ outcome*)	Sub-code under Gaps. This code will capture gaps that have contributed to the dearth in research on racial dimensions that are more tangible and material in nature, as compared to the more attitudinal and intangible gaps.	(Creutzfeldt and Gill 2021), (Hodges, Schmidt and Becker 2022), (UKERC 2022)
2.3.	Methods (*methods*)	Sub-code under Gaps. This looks at some of the methodological challenges that currently exist in terms of being able to address racial considerations in energy demand research in a more robust manner	
2.3.1.	Intersectionality (*methods*)	Sub-code under Methodology. This code captures reflections how because race is not just a single attribute to meaningfully explain changes, it needs to be	(Blakelock 2021)

Level	Code Name	Description	References
		associated with other characteristics like gender and class, it becomes challenging to articulate this meaningfully.	
2.3.2.	No precedence	Sub-code under Methodology. Captures gaps that have been identified in the sense of methodological challenges experienced because there has been no precedence in attempting to consider such attributes (or datasets) therefore requiring methodological creativity.	(Ahmadzadeh 2021), (British Future 2021), (Mistlin 2021)
2.3.3.	Policy	Sub-code under Methodology. The way policies are structured or disincentivise the availability of certain data. E.g. suppliers aren't expected to collect data on race, and hence they don't. Sometimes, because policies don't explicitly have race as a characteristic, this data doesn't get captured.	(Bednar and Reames 2020), (Raslan and Ambrose 2022), (UKERC 2022)
2.3.4.	Trust & participation (*People/ engagement*)	Sub-code under Methodology. Absence of trust to identify as a racial minority thus being a barrier to addressing the challenges, and providing relevant data to develop the evidence base.	(Blakelock 2021), (UKERC 2022), Kapadia (2021)
2.4.	Mindset (*methods*)	Sub-code under Gaps. Is there a fundamental mindset problem that makes it difficult to recognise racial injustice in the energy system and more systemically, that needs to be considered? One way to ameliorate this gap, can be through the political education approach that exists.	
2.4.1.	Discomfort (*methods*)	Sub-code under Mindset. This code captures discomfort that people (especially in power) experience when talking about colonialism or racism, and therefore the issue gets invisibilised.	
2.4.2.	Diversity (*People/ engagement*)	Sub-code under Mindset. Code to capture reflections on lack of diversity in key decision-making areas that leads to a gap in the research.	(EPSRC 2022)
2.4.3.	Education, Awareness (*methods*)	Sub-code under Mindset. The idea that imperialism was a benign development project, as is articulated in education (and might need to be unlearnt)	
2.4.4.	Technical (*theme/ outcome*)	Sub-code under Mindset. This code captures information on the perception that because energy is considered as a technical topic, the people and socio-cultural dimensions that inform it, do not get sufficiently captured.	
2.4.5.	Urgency	Sub-code under Mindset. This code captures information on how urgency is used almost as an excuse to not consider the social justice implications, and aids in deprioritising the need for more focus on marginalisation.	
2.5.	Structural (*theme/ outcome*)	Sub-code under Gaps. This code captures the systemic challenges in the way the existing energy system is structured that might hinder a more equitable pathway.	(Newell 2005), (Newell 2021),

3. Workshop design and objectives

Objectives of the workshop and expectation:

- Present status of the research, and emerging findings (based on literature review and qualitative analysis of 27 semi-structured interviews)
- Obtain feedback on the research, and include participants who the research process might have been unable to speak to until then. The workshop will be recorded, and the discussion will be used as an input to finalise the findings
- It will also be an invitation for participants to be involved in future opportunities to take the research and emerging recommendations forward

When? 26th April, 2023

Medium: Online

Participants: 45 from academic, research and practitioner roles in the UK and Europe, working on energy and social justice related issues

Emerging findings from the thematic analysis were shared with the 45 workshop participants, who were then asked to pick one of the following groups to participate in break-out group discussions, to finalise the findings. These groups are aligned with the themes identified during the thematic analysis:

- Identifying and articulating challenges in existing energy demand research on incorporating racial justice principles/ Why have we not been able to make racial justice more explicit in energy demand research?
- How do we build evidence of racialisation across energy-related areas such as built environment, health, transport, etc.?
- How can dimensions of energy demand research, including energy cultures and practice, energy advice, intermediaries, etc. understand racialisation better?
- How do we practise and sustain anti-racist research in energy demand?
